# 1110. Why has Utilization of Novel Gram-negative Antibiotics been Sluggish in U.S. Hospitals? A Retrospective Cohort Study to Inform Future Antibiotic Development and Market Entry Rewards

**DOI:** 10.1093/ofid/ofad500.083

**Published:** 2023-11-27

**Authors:** Jeffrey R Strich, Ahmed Ullah Mishuk, Alexander Lawandi, Guoqing Diao, Sarah Warner, Willy Li, Cumhur Y Demirkale, Alex Mancera, Morgan Walker, Christina Yek, Maniraj Neupane, Nathaniel De Jonge, Sameer S Kadri

**Affiliations:** Clinical Center, National Institutes of Health, Bethesda, Maryland; Critical Care Medicine Department, National Institutes of Health Clinical Center, Bethesda, Maryland; Critical Care Medicine Department, Clinical Center, National Institutes of Health, Bethesda, MD, Montreal, Quebec, Canada; George Washington University Milken Institute School of Public Health, Washington, District of Columbia; Critical Care Medicine, National Institutes of Health Clinical Center, Bethesda, Maryland; Critical Care Medicine Department, Clinical Center, National Institutes of Health, Bethesda, MD, 4. Department of Pharmacy, Clinical Center, National Institutes of Health, Bethesda, MD, Bethesda, Maryland; National Institutes of Health, Bethesda, MD; Critical Care Medicine Department, Clinical Center, National Institutes of Health, Bethesda, MD, 2. Critical Care Medicine Branch, National Heart Lung and Blood Institute, Bethesda, MD, Bethesda, Maryland; Critical Care Medicine Department, Clinical Center, National Institutes of Health, Bethesda, MD, 2. Critical Care Medicine Branch, National Heart Lung and Blood Institute, Bethesda, MD, Bethesda, Maryland; National Institute of Allergy and Infectious Diseases, Bethesda, Maryland; Critical Care Medicine Department, Clinical Center, National Institutes of Health, Bethesda, MD, 2. Critical Care Medicine Branch, National Heart Lung and Blood Institute, Bethesda, MD, Bethesda, Maryland; Critical Care Medicine Department, Clinical Center, National Institutes of Health, Bethesda, MD, 2. Critical Care Medicine Branch, National Heart Lung and Blood Institute, Bethesda, MD, Bethesda, Maryland; National Institutes of Health Clinical Center, Bethesda, Maryland

## Abstract

**Background:**

Sluggish uptake of novel gram-negative antibiotics has jeopardized their future development and supply potentially risking patient lives. Understanding underutilization patterns might better inform unmet needs and market entry rewards. We studied patterns and drivers of use of recently U.S. FDA-approved novel gram-negative agents in patients with gram-negative infections (GNI) displaying difficult-to-treat resistance (DTR; resistance to all first line B-lactams including carbapenems, and fluoroquinolones).

**Methods:**

Using the all-payer PINC-AI database, quarterly percent change in the post-approval use of seven gram-negative agents and secular trends in colistin use were calculated. For inpatients with DTR GNI at microbiology reporting hospitals, we compared novel vs older/reserve agent use by infection site and pathogen. At hospitals that use novel agents, generalized linear mixed models with weighting identified patient and hospital factors associated with the use of any novel (vs older/reserve) agents as targeted therapy against DTR GNI.

**Results:**

At 619 hospitals, while colistin use declined between 2016Q1 and 2021Q2, post-approval use grew variably for each novel agent (Fig1); compared to the first novel anti–*Klebsiella pneumoniae* carbapenemase (KPC) agent ceftazidime-avibactam (approved 2015), usage was relatively sluggish for subsequently approved anti-KPC-agents meropenem-vaborbactam and imipenem-cilastatin-relebactam and zero for plazomicin. At 302 microbiology reporting hospitals, 44.4% of 2830 overall DTR cases including 70.2% of 627 DTR-*A. baumannii* cases received only older/reserve agents (Fig2). The odds of use of novel (vs older/novel) agents were greater in patients displaying hemodynamic instability and higher comorbidity burden, but similar across race categories and insurance status, and lower at Western(vs Midwestern) hospitals (Table1).

**Figure:**
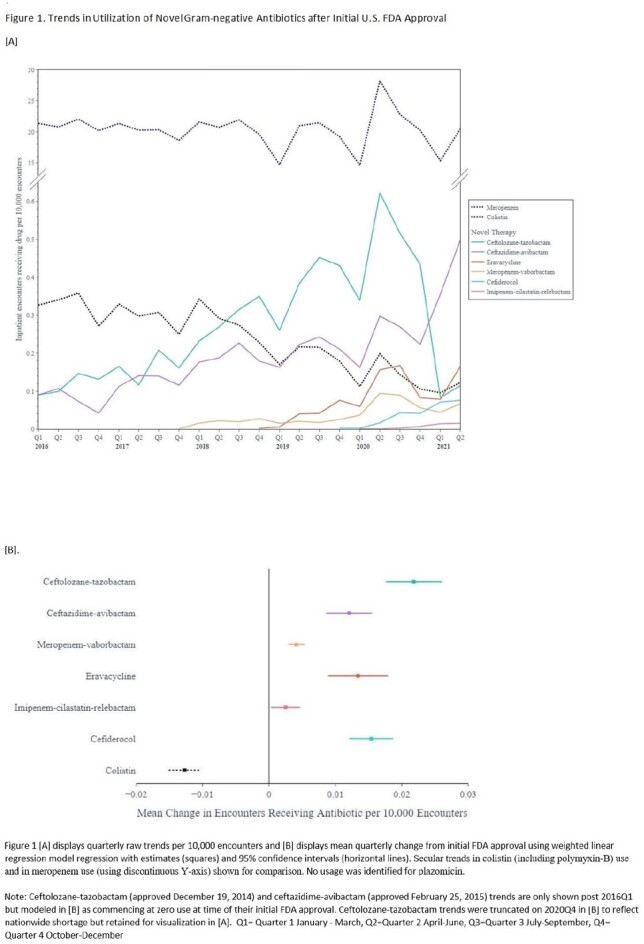

**Figure:**
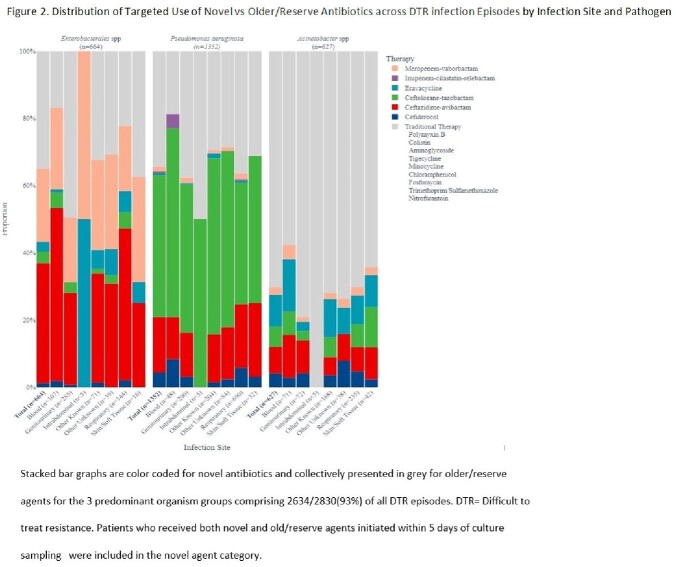

**Figure:**
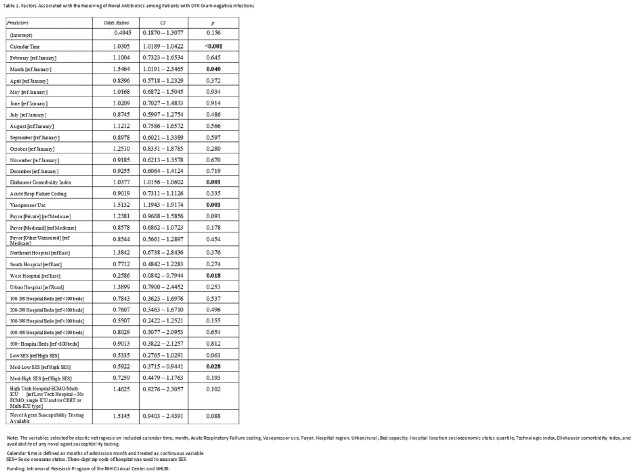

**Conclusion:**

Nearly half the patients in US hospital with DTR-GNIs are still receiving targeted therapy with older/reserve agents. While the value of novel agents is indicated by clinicians preferring them over older/reserve agents in sicker patients, future agents with distinctly novel mechanisms and activity against DTR-*A. baumannii* are needed to bridge the utilization gap.

**Disclosures:**

**Morgan Walker, MD**, Cytovale: Advisor/Consultant

